# A signature based on circadian rhythm-associated genes for the evaluation of prognosis and the tumour microenvironment in HNSCC

**DOI:** 10.1038/s41598-024-57160-5

**Published:** 2024-03-31

**Authors:** Changqian Wang, Xiang Liu, Pengkhun Nov, Lilin Li, Chunhui Li, Xuejiao Liao, Luyao Li, Kunpeng Du, Jiqiang Li

**Affiliations:** 1 Department of Radiation Oncology, Oncology Center, Zhujiang Hospital of Southern Medical University, Guangzhou, 510282 Guangdong Province China; 2 Department of Oncology, Shenzhen Hospital of Southern Medical University, Shenzhen, China

**Keywords:** HNSCC, Circadian rhythm, Tumour microenvironment, Metabolism, Immunity, Functional clustering, Head and neck cancer

## Abstract

The morbidity and mortality rates of head and neck squamous cell carcinoma (HNSCC) remain high worldwide. Therefore, there is an urgent need to identify a new prognostic biomarker to guide the personalized treatment of HNSCC patients. Increasing evidence suggests that circadian rhythm genes play an important role in the development and progression of cancer. We aimed to explore the value of circadian rhythm genes in predicting prognosis and guiding the treatment of HNSCC. We first obtained a list of circadian rhythm genes from previous research. The sequencing data were retrieved from The Cancer Genome Atlas (TCGA) and Gene Expression Omnibus (GEO) databases. Finally, univariate Cox proportional hazard analysis, least absolute shrinkage and selection operator (LASSO) regression, and multivariate Cox proportional hazard analysis were performed to develop a prognostic signature (Circadian Rhythm-Related Gene Prognostic Index, CRRGPI) consisting of nine circadian rhythm genes. The signature exhibited good performance in predicting overall survival. Patients with low CRRGPI scores had lower metabolic activities and an active antitumour immunity ability. Additionally, a clinical cohort was used to further evaluate the ability of the CRRGPI to predict the efficacy of immune checkpoint inhibitors. In conclusion, the novel circadian rhythm-related gene signature can provide a precise prognostic evaluation with the potential capacity to guide individualized treatment regimens for HNSCC patients.

## Introduction

In recent years, with the widespread use of immune checkpoint inhibitors (ICIs) in tumour therapy and the significantly prolonged survival time of patients, programmed death-1 (PD-1) inhibitors have been approved for the treatment of head and neck squamous cell carcinoma (HNSCC) patients with recurrence or metastasis after platinum-based chemotherapy^1^. However, the overall response rate of HNSCC patients to ICIs is low, and only a small number of patients can benefit from them. Therefore, there is an urgent need to identify a new prognostic biomarker to guide the personalized treatment of HNSCC patients.

The circadian rhythm is the timing system of the body that involves a variety of processes, including the sleep-awakening cycle, eating-fasting cycle, and activity-rest cycle. These processes coordinate the behaviour and physiology of all organs to achieve systemic homeostasis. Disordered circadian rhythms lead to body dysfunction, such as abnormal cell proliferation, increased numbers of gene mutations, and resistance to apoptosis, which increase the incidence of many diseases^2^. An increasing number of studies have shown that a disordered circadian rhythm participates in the occurrence and development of many cancers, such as breast cancer, colorectal cancer, and lung cancer, through different mechanisms^2–5^. These mechanisms include the imbalance of immune cells, the release of cytokines, the inflammatory response, DNA damage, endocrine disorders, and so on^2^. Yang et al. reported that the biological clock gene *PER1* can regulate the circadian rhythm of oral squamous cell carcinoma (OSCC) cell proliferation and apoptosis by altering the circadian rhythm characteristics of the protein kinase B (AKT)/mammalian target of rapamycin (mTOR) pathway^6^. Moreover, the efficacy of HNSCC treatment partly depends on the delivery time of radiotherapy^7^. Therefore, understanding the characteristics of circadian rhythm genes may provide a new strategy for the treatment of cancer.

Currently, there are few studies on the role of circadian rhythm genes in HNSCC. According to previous studies, we speculate that the characteristics of circadian genes are associated with the prognosis of HNSCC patients. In this study, we used bioinformatics research methods to construct a prognostic signature (Circadian Rhythm-Related Gene Prognostic Index, CRRGPI) consisting of 9 circadian rhythm genes. We found that the CRRGPI could better distinguish the clinical outcome of HNSCC patients and that there were differences in tumour stemness, metabolic activity, and immune characteristics among patients with different CRRGPI scores. The CRRGPI can also predict the possible clinical benefits of patients receiving ICIs.

## Materials and methods

### Patients and datasets

The RNA sequencing (RNA-seq) data and clinicopathologic information of 546 HNSCC samples, including 502 tumour samples and 44 normal samples, were obtained from The Cancer Genome Atlas (TCGA) database through the Genomic Data Commons Data Portal (https://portal.gdc.cancer.gov/). The RNA-seq data of 270 HNSCC tumour samples (GSE65858), corresponding survival information, and sequencing platform annotation information (GPL570) were downloaded from the Gene Expression Omnibus (GEO) database (https://www.ncbi.nlm.nih.gov/geo/).

### Identification of circadian rhythm-related differentially expressed genes

To obtain the circadian rhythm-related differentially expressed genes (CRRDEGs), we first obtained the differentially expressed genes (DEGs) (false discovery rate (FDR) < 0.05, |log2 fold change (FC)|> 1.0) between the 502 tumour samples and 44 normal samples from TCGA by using the limma package of R software. Then, the DEGs were intersected with the circadian rhythm-related gene lists, and CRRDEGs were identified. Volcano plots and hot plots of DEGs and CRRDEGs were plotted using the ggplot2 package of R.

### Development and validation of the Circadian Rhythm-Related Gene Prognostic Index (CRRGPI)

The NMF and survival packages of R were used to identify the best classification according to the expression of circadian rhythm-related genes and the overall survival (OS) time of HNSCC patients. After determining the best classification, the whole dataset was randomly split into a training cohort (351/499, 70%) and a test cohort (148/499, 30%). The circadian rhythm-related genes were subjected to univariate Cox regression assessment to identify those genes linked to HNSCC OS. Only those genes that were statistically significant (*P* < 0.05) were included in the least absolute shrinkage and selection operator (LASSO) regression analysis. Then, the prognostic signature was developed using multivariate regression. Thus, we calculated the CRRGPI score by combining the expression of the included genes weighted by the linear regression and coefficients. The CRRGPI score was calculated by using the following equation: CRRGPI = Exp1 ∗ Coe1 + Exp2 ∗ Coe2 + Exp3 ∗ Coe3 + …Expi ∗ Coei.

The training and test cohorts were divided into the high-CRRGPI group and low-CRRGPI group according to the median CRRGPI. The ability of the CRRGPI to discriminate the prognosis of patients was evaluated by the Kaplan‒Meier survival curve and the log-rank test.

In addition, to verify the independent prognostic role of the CRRGPI, the CRRGPI and other clinicopathological factors were included in univariate and multivariate Cox regression analyses.

### Identification of biological characteristics of the two Circadian Rhythm-Related Gene Prognostic Index groups

To explore the biological characteristics of the two CRRGPI groups, we first used the limma package of R to identify the DEGs between the high-CRRGPI and low-CRRGPI groups. Then, gene set enrichment analysis (GSEA) was performed to determine the signalling pathways enriched in different CRRGPI subgroups based on the Kyoto Encyclopedia of Genes and Genomes (KEGG) gene sets (c2.cp.kegg.v7.4) by using the clusterProfiler (version 3.14.3) package of R [*P* < 0.05, FDR < 0.25]. Gene Ontology (GO) and KEGG analyses^8–10^ were performed in the Sangerbox Web^11^ to explore the biological functions and processes in which CRRDEGs were involved.

The data on genetic alterations in HNSCC were obtained from the TCGA GDC data portal, and the Maftools package was used to analyse the number and categories of gene mutations in the two CRRGPI groups. The tumour mutation burden (TMB) of each sample was also calculated by the Maftools package of R.

To compare the metabolic characteristics between the two CRRGPI groups, we downloaded and sorted the relevant metabolic gene sets from the Gene Set Variation Analysis (GSVA) database and scored each sample according to the genes by the single-sample GSEA (ssGSEA) algorithm. A t test was used to compare the differences between the two groups. The association of the RNA stemness score (RNAss) and DNA stemness score (DNAss) with the CRRGPI was determined using the R packages limma and ggplot2. Spearman’s test was also used during the analysis.

### Comprehensive analysis of immune characteristics and the benefit of immune checkpoint inhibitors in the different Circadian Rhythm-Related Gene Prognostic Index groups

There are many immune steps in the antitumour immune cycle, including the release of tumour antigens, the presentation of tumour antigens, the initiation and activation of immune cells, the transport of immune cells to the tumour, the infiltration of immune cells into the tumour, the recognition of T cells by cancer cells and the killing of cancer cells. To further compare whether there were differences in antitumour immunity among the different CRRGPI subgroups, we collected a list of genes representing these seven antitumour immune steps from the Tracking Tumour Immunophenotype (TIP) website, which is shown in the Supplementary Table 2. ssGSEA, an algorithm that can calculate the absolute enrichment of a given gene set according to the gene expression value to indirectly reflect the activity or enrichment of a given gene set, was used to score the seven immune processes in the antitumour immune cycle of 499 HNSCC tumour specimens and evaluate the tumour characteristics of the high-CRRGPI and low-CRRGPI groups. In addition, to further evaluate the difference in immune cell infiltration between different CRRGPI groups, we used the CIBERSORT algorithm to quantitatively analyse the relative abundance of 22 immune cell types in 499 HNSCC tumour samples, where 0 was the minimum value and 1 was the highest value for each immune cell type.

The immune status of different CRRGPI groups was compared. The ESTIMATE algorithm, a tool using gene expression data to predict tumour purity, stroma, and infiltration of immune cells in tumour tissue, was used to calculate three scores according to the ssGSEA: (1) stromal score (capturing the presence of stroma in tumour tissue), (2) immune score (representing the infiltration of immune cells in tumour tissue), and (3) ESTIMATE score (inferred tumour purity).

Then, we assessed the TIDE score, including dysfunctional cytotoxic T cells, exclusionary cytotoxic T cells, interferon-gamma (IFNG) score, M2 tumour-associated macrophages (M2-TAMs), cancer-associated fibroblasts (CAFs), and myeloid-derived suppressor cell (MDSC) infiltration, with the TIDE algorithm^12^ to compare the antitumour and tumour immune escape abilities of the two CRRGPI groups.

### Immunotherapy response and drug sensitivity analysis in different Circadian Rhythm-Related Gene Prognostic Index groups

To explore the prognostic value of the CRRGPI for patients undergoing immunotherapy, an immunotherapeutic cohort was used in our study: advanced urothelial cancer patients with the intervention of atezolizumab, an anti-PD-L1 antibody (IMvigor210 cohort)^13^. Clinical information and gene expression data were extracted from the IMvigor210 dataset (http://research-pub.gene.com/IMvigor210CoreBiologies). The R package pRRophetic^14^ and the CellMiner database were used to predict chemotherapeutic drug sensitivity in different CRRGPI groups.

### Statistical analysis

We completed analyses using software R 4.1.3 and its suitable packages. The Wilcoxon test was used to analyze the expression differences between the high-CRRGPI and low-CRRGPI. Survival analysis was conducted through log-rank test and presented as Kaplan–Meier curve to compare survival between subgroups. Statistical significance was set as two-sided *P* < 0.05.

## Results

### Identification of circadian rhythm-related genes

**Figure 1:**
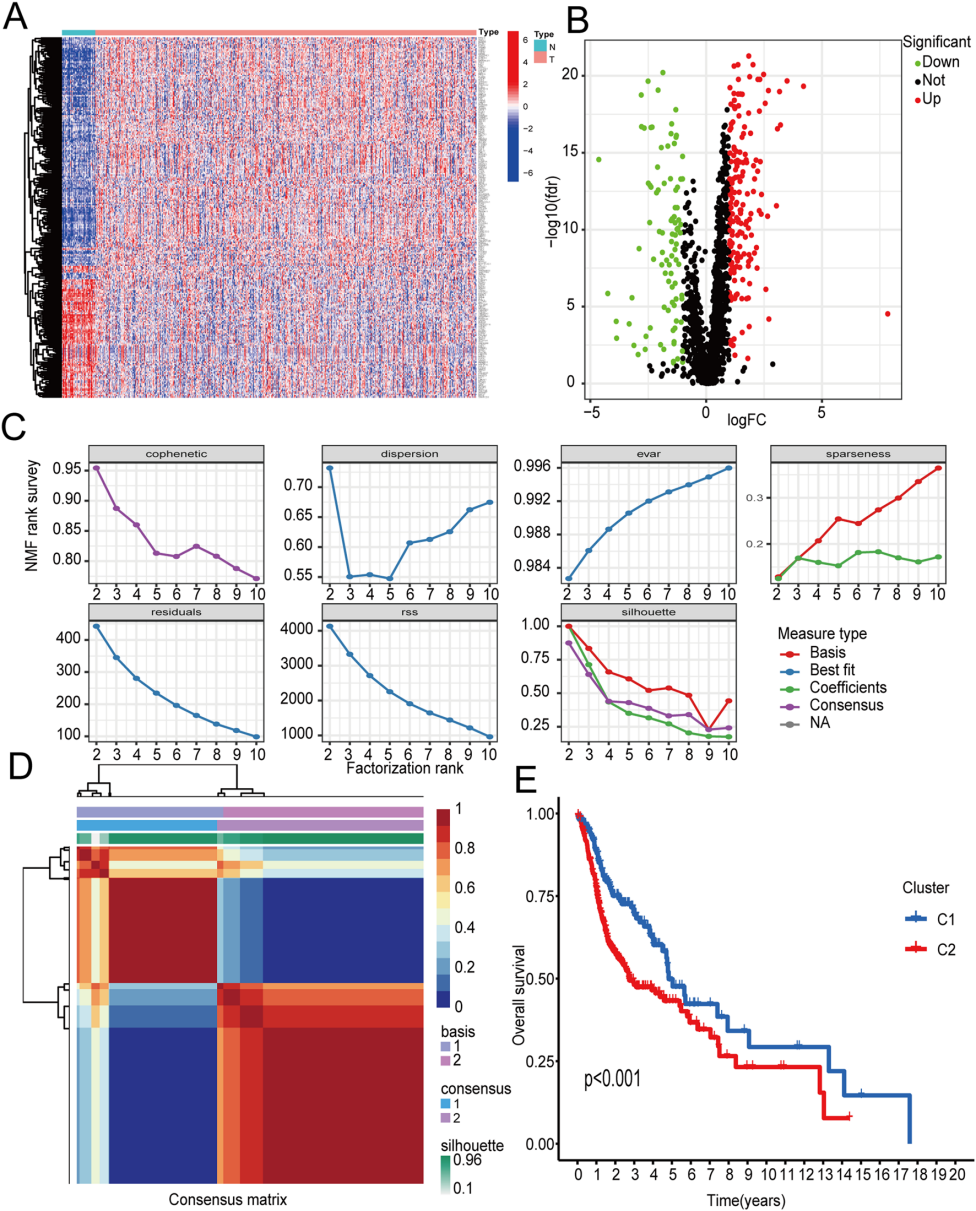
Identification of circadian rhythm-related genes and results of NMF-based tumour typing. (**A**) Visualization of RNA expression and (**B**) differentially expressed RNA between tumour and normal tissues (red and green dots indicate upregulated and downregulated RNAs, respectively). (**C**) The relationship between cophenetic, dispersion, and silhouette coefficients, and (**D**) the consensus map with respect to the number of clusters. (**E**) K–M survival curves of modification subtypes.

The data of 546 HNSCC samples (44 normal and 502 tumours) downloaded from the TCGA database were analysed. According to the expression level of the circadian rhythm genes^15^ in 546 HNSCC samples, a total of 268 circadian rhythm-related genes were obtained by difference analysis between normal and tumour samples (Fig. 1A). The volcano map visualizes the up- and downregulated circadian rhythm-related genes (Fig. 1B).

### Construction of the CRRGPI and external verification

According to the expression of these 268 circadian rhythm-related genes in the HNSCC samples, the HNSCC patients were divided into multiple clusters by the NMF algorithm. According to the results of cophenetic, dispersion, residuals, and silhouette in the NMF rank survey and the heatmap (Fig. 1C,D), the HNSCC cohort was divided into Cluster 1 and Cluster 2. Furthermore, the Kaplan–Meier (K‒M) overall survival curve showed that the prognosis of patients in Cluster 1 was better than that of patients in Cluster 2 (Fig. 1E) (*P* < 0.001), which indicated that two clusters were the best categorization for distinguishing the prognosis of HNSCC patients.

**Figure 2:**
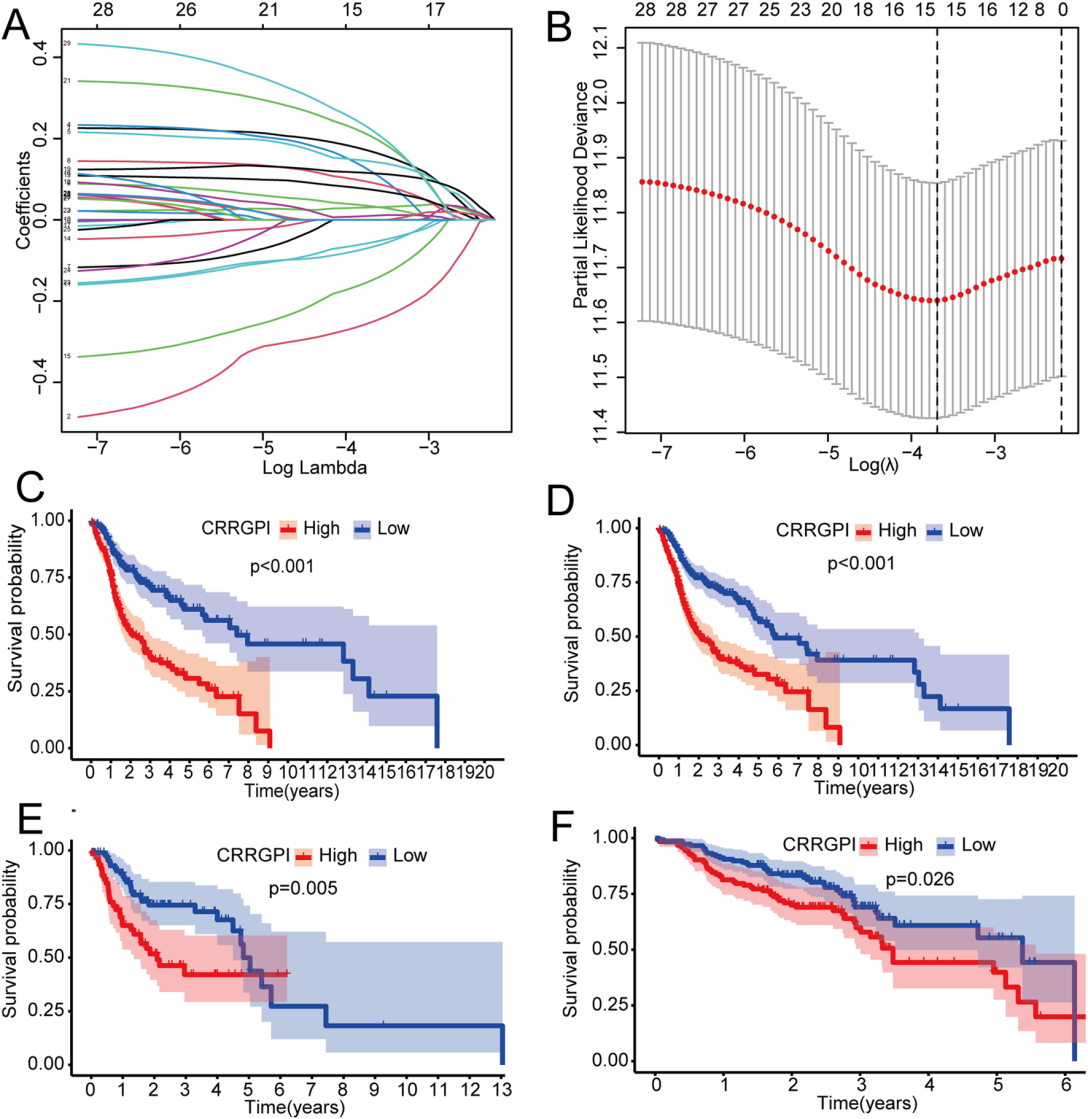
CRRGPI construction and validation. (**A**) LASSO coefficients of prognostic gene sets. (**B**) Threefold cross-validation for the selection of prognostic gene sets in LASSO regression. CRRGPI was constructed based on LASSO regression analysis and validated in the training cohort (**C**), whole TCGA cohort (**D**), test cohort (**E**), and GSE65858 cohort (**F**). CRRGPI, Circadian Rhythm-Related Gene Prognostic Index.

To construct a prognostic signature for HNSCC patients, 499 patients with complete clinical data were randomly divided into training and test cohorts at a ratio (n:m) of 7:3. There were 351 patients in the training cohort and 148 patients in the test cohort. In the training cohort, 29 circadian rhythm genes related to prognosis were obtained by univariate Cox regression analysis, and 15 genes (*ADA*, *ICOS*, *SEC61G*, *ALG3*, *CBX3*, *STC1*, *BASP1*, *CYP4X1*, *DCBLD1*, *DHCR7*, *EZH2*, *OLR1*, *PLEKHA6*, *PTPRN2*, and *STARD4*) were further selected by LASSO regression analysis (Fig. 2A,B). Finally, the CRRGPI was constructed using 9 circadian rhythm genes (*ADA*, *ICOS*, *ALG3*, *STC1*, *CYP4X1*, *EZH2*, *OLR1*, *PLEKHA6*, and *STARD4*) by multivariate Cox regression analysis. The CRRGPI formula was as follows: CRRGPI = ADA*0.274416 + ICOS*-0.408837 + ALG3*0.289390 + STC1*0.142689 + CYP4X1*-0.139852 + EZH2*-0.238670 + OLR1*0.151689 + PLEKHA6*0.296361 + STARD4*0.420515. According to the CRRGPI scores of the samples using 50% as the cut-off, the samples in the test and training cohorts were divided into a high-CRRGPI group and a low-CRRGPI group. In addition, data from the training cohort were used to evaluate the value of CRRGPI in predicting the survival of patients. As shown in Fig. 2C, the OS of patients in the high-CRRGPI group was worse than that in the low-CRRGPI group (*P* < 0.001), indicating that CRRGPI is a poor prognostic factor. This effect was further verified in the test cohort (*P* < 0.005) and the whole TCGA cohort (*P* < 0.001) (Fig. 2D,E). According to the CRRGPI, we scored each sample of the GEO dataset and then analysed the OS of the patients (Fig. 2F). The results were consistent with the TCGA cohort, and the OS of patients in the high-CRRGPI group was worse (*P* = 0.026), which further confirms the stability of the CRRGPI we developed.

### Assessment of CRRGPI as an independent HNSCC prognostic factor

**Figure 3:**
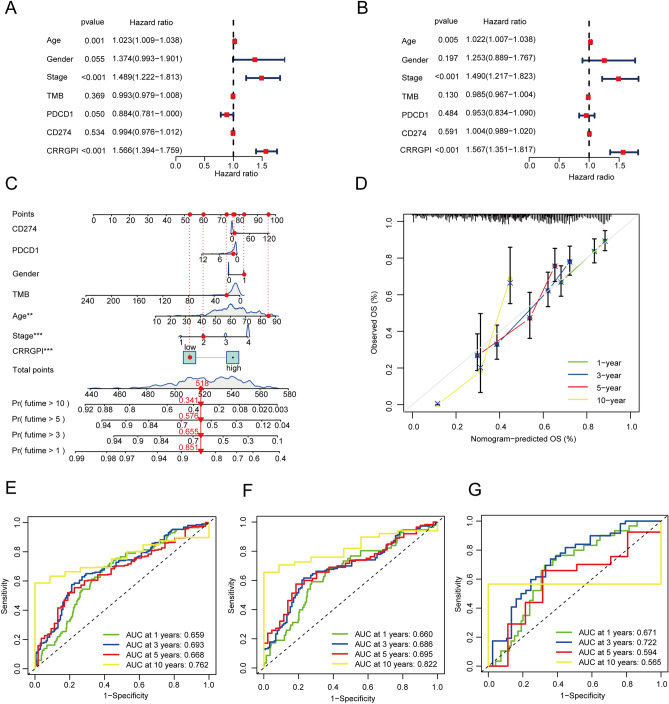
Assessment of CRRGPI as an independent HNSCC prognostic factor. Assessment Of CRRGPI As Independent HNSCC Prognostic Factors for OS. (**A**, **B**) The forest map shows the CRRGPI and the clinical variables by univariate Cox regression analysis and multivariate Cox regression analysis. (**C**) The visualization of overall survival prediction with the CRRGPI and clinical variables in the nomogram (*P < 0.05, **P < 0.01, ***P < 0.001). (**D**) The calibration curves of the nomogram. (**E**, **F**, **G**) The receiver operating characteristic (ROC) curves of the CRRGPI of patients in the whole TCGA cohort, training cohort, and test cohort, AUC area under curve.

Univariate Cox regression, multivariate Cox regression, and receiver operating characteristic (ROC) analysis were employed to determine whether the CRRGPI has prognostic value in HNSCC overall survival independent of clinicopathological indicators such as age, pathological stage, sex, PDCD1, CD274, and TMB. The hazard ratios (HRs) and 95% confidence intervals (CIs) of the CRRGPI were 1.566 and 1.394–1.759 in the univariate Cox regression assessment (*P* < 0.001) and 1.567 and 1.351–1.817 in the multivariate Cox regression assessment (*P* < 0.001) (Fig. 3A–B). The CRRGPI has also prognostic value in HNSCC progression-free survival (Supplementary Fig. 1). Moreover, we constructed a nomogram including age, pathological stage, sex, PDCD1, CD274, TMB, and CRRGPI for prognosis prediction in patients with HNSCC (Fig. 3C). The calibration curves of the nomogram revealed that the predicted and observed survival probabilities of one-year, three-year, five-year, and ten-year OS had a high degree of consistency (Fig. 3D). Finally, ROC analysis was performed to assess the predictive power of the CRRGPI. The area under the time-dependent ROC curve (AUC) for all HNSCC patients was 0.659, 0.693, 0.668, and 0.762 for one-year, three-year, five-year, and ten-year OS (Fig. 3E), respectively. The AUCs at 1 year, 3 years, 5 years, and 10 years for the training cohort (Fig. 3F) and test cohort (Fig. 3G) were 0.660, 0.686, 0.695, and 0.822 and 0.671, 0.722, 0.654, and 0.656, respectively. The CRRGPI was also verified to have good predictive performance in the GEO dataset (Supplementary Fig. 2A). Furthermore, to verify the superior predictive ability of the CRRGPI, we compared it with two commonly used predictive biomarker Tumor Immune Dysfunction and Exclusion (TIDE) scores^12^ and tumor inflammation signature (TIS)^16^. The ROC curve results showed that the CRRGPI had a better predictive ability than the two gene sets previously reported in the literature (Supplementary Fig. 2B). These results demonstrated that the CRRGPI performed well in predicting the OS of HNSCC patients.

### Subgroup survival analysis

**Figure 4:**
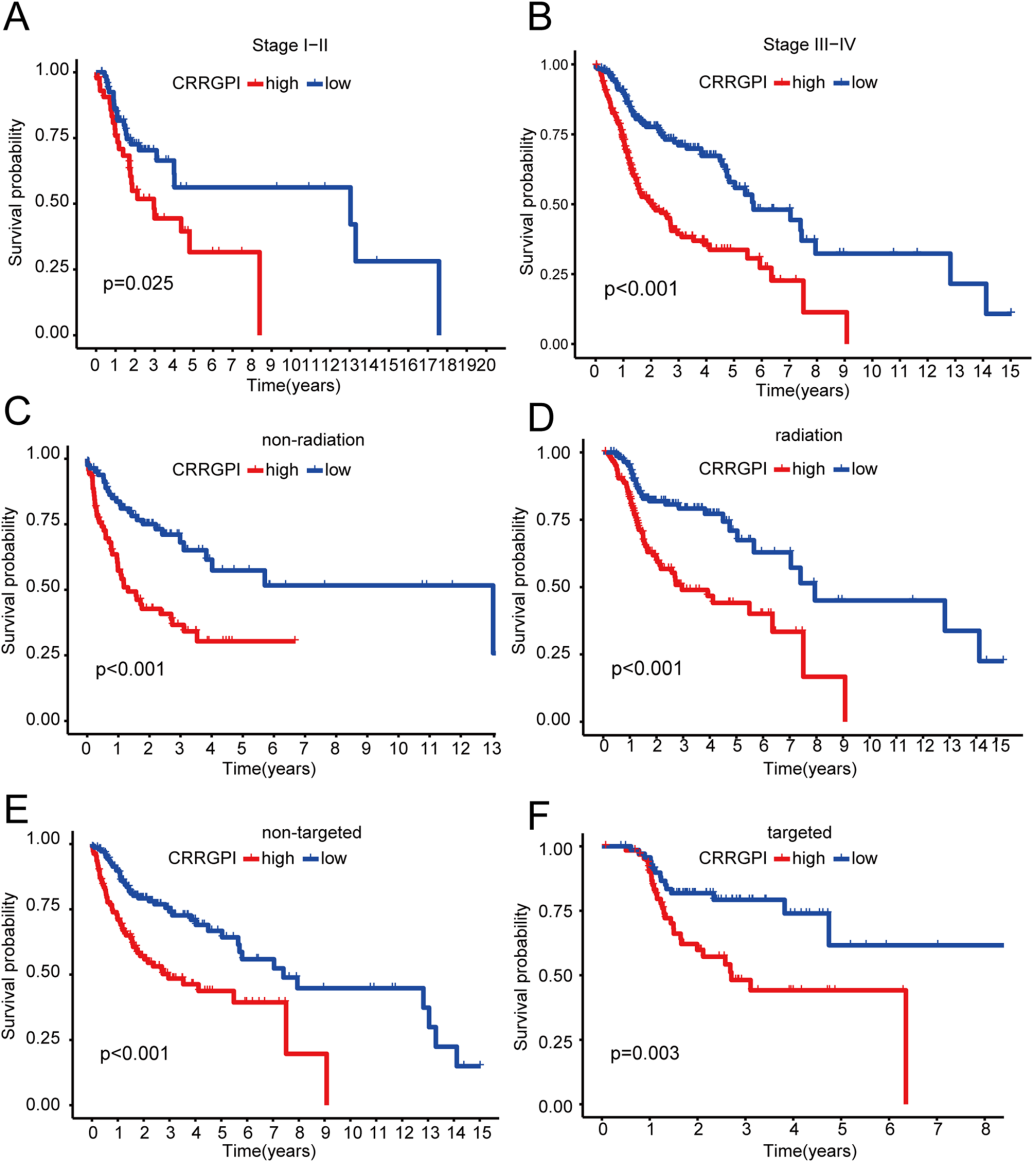
Subgroup survival analysis. Kaplan‒Meier plots of the high-CRRGPI and low-CRRGPI subtypes of HNSCC in 6 subgroups of (**A**) patients with stage I-II, (**B**) patients with stage III–IV, (**C**) patients without radiation, (**D**) patients with radiation, (**E**) patients without targeted therapy, and (**F**) patients with targeted therapy. CRRGPI, Circadian Rhythm-Related Gene Prognostic Index.

Additionally, according to the clinicopathological characteristics of patients, a subgroup survival analysis was performed. The analysis of K‒M survival curves showed significant differences in survival for different ages, sexes (*P* < 0.001) (Supplementary Fig. 3), and stages (*P* < 0.05) (Fig. 4A,B). Moreover, the patients were divided into radiation therapy, nonradiation therapy, molecular targeted therapy, and nonmolecular targeted therapy subgroups according to the previous therapy history of patients. Among the four subgroups, patients in the high-CRRGPI group had worse OS than those in the low-CRRGPI group (*P* < 0.05) (Fig. 4C–F). This suggested that the CRRGPI had an excellent ability to distinguish patient outcomes in all four subgroups.

### Molecular characteristics of different CRRGPI groups

**Figure 5:**
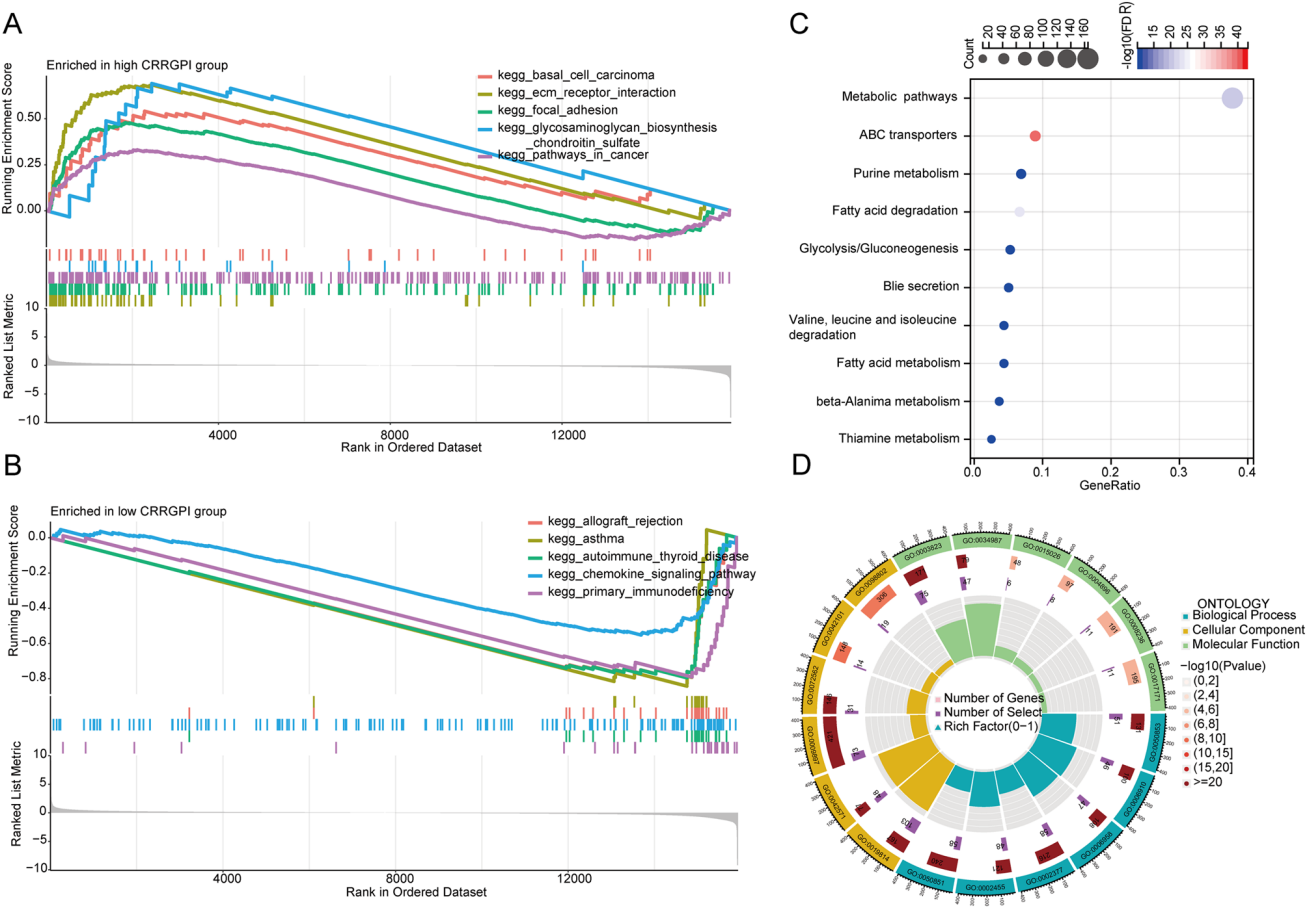
Functional annotation of differentially expressed genes. (**A**) GSEA analysis in the high-CRRGPI and (**B**) low-CRRGPI groups. (**C**) Kyoto Encyclopedia of Genes and Genomes (KEGG) pathways and (**D**) Gene Ontology (GO) functions enriched in the differentially expressed genes between high-CRRGPI and low-CRRGPI groups.

To explore the molecular characteristics of the different CRRGPI groups, the GSEA algorithm was used to evaluate the enrichment of DEGs between these groups. Based on the KEGG database, our results show that the genes enriched in the high-CRRGPI group were mainly associated with basal cell carcinoma, extracellular matrix (ECM) receptor interaction, focal adhesion, glycosaminoglycan biosynthesis chondroitin sulfate,, and cancer pathway (Fig. 5A), while those enriched in the low-CRRGPI group were mainly involved in allograft rejection, asthma, autoimmune thyroid disease, chemokine signalling pathways, and primary immunodeficiency (Fig. 5B). Details of the GSEA data can be found in supplementary Table 1. In addition, to explore the differences in cell function between the high-CRRGPI group and the low-CRRGPI group, we carried out KEGG and GO functional pathway enrichment analyses to analyse the DEGs between the two groups (Fig. 5C,D).

**Figure 6:**
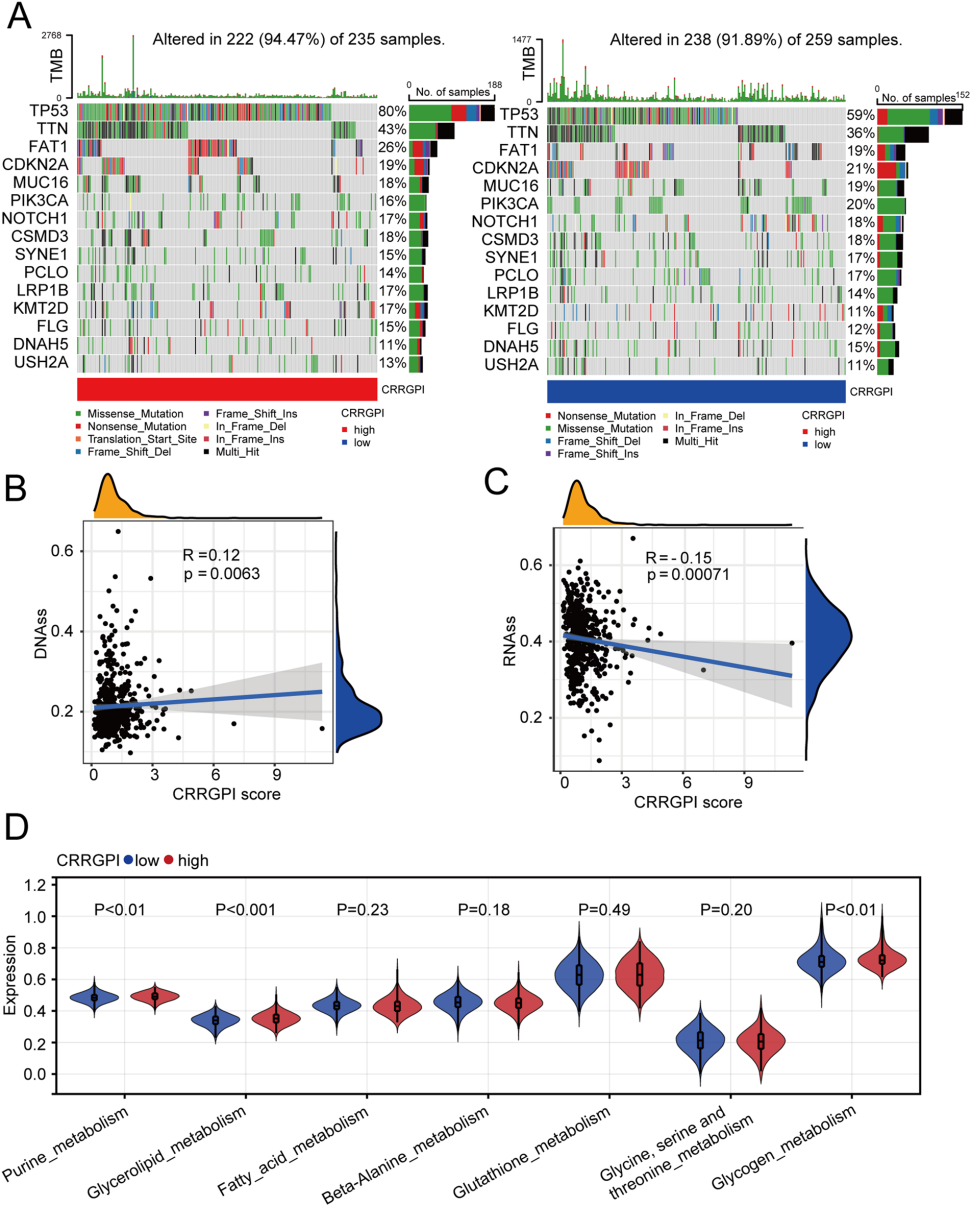
Biological characteristics of different CRRGPI groups. (**A**) Top 20 mutated genes in the different CRRGPI subgroups. Mutated genes (rows) are ordered by mutation rate; samples (columns) are arranged to emphasize mutual exclusivity among mutations. The rightmost label shows the mutation percentage, and the top shows the overall mutation number of the patients. The coloured squares indicate the mutation type. (**B**, **C**) Correlation analysis of CRRGPI with DNAss and RNAss. (**D**) The scores of common metabolic pathways of the different CRRGPI subgroups. DNAss, DNA stemness score; RNAss, RNA stemness score. CRRGPI, Circadian Rhythm-Related Gene Prognostic Index.

Moreover, to compare immunological features between different groups, we analysed TMB in different CRRGPI groups. The results showed that there was no significant difference in TMB between the two CRRGPI groups (94.47% vs. 91.89%, *P* = 0.088), but we observed that the gene with the highest mutation frequency in both groups was TP53 (Fig. 6A). A missense mutation was the most frequent type, and the TP53 mutation frequency of the high-CRRGPI group was higher than that of the low-CRRGPI group (80% vs. 59%) (Fig. 6A). In HNSCC, there is a high frequency of loss-of-function mutations in the tumour suppressor gene p53, which leads to the generation of more neoantigens and activates the antitumour immune response, resulting in increased infiltration of immune cells into the tumour. These antitumour responses can improve the therapeutic outcomes of HNSCC patients^17,18^.

Furthermore, due to rapid tumour growth, tumour cells may lose their differentiation phenotype and exhibit stem-like characteristics. Some studies have reported that stemness properties are largely responsible for tumour recurrence, metastasis, and chemoresistance^19^. We further explored the correlation between the CRRGPI and tumour stemness. Stemness scores based on mRNA expression (RNAss) and DNA methylation (DNAss) were utilized to measure the correlation between tumour stemness and CRRGPI score (Fig. 6B,C). According to KEGG functional analysis, we found that the DEGs between the high- and low-CRRGPI groups were mainly involved in metabolic pathways. Next, we compared the metabolic characteristics of the high-CRRGPI group and the low-CRRGPI group. Our results showed that purine metabolism, glycerolipid metabolism, and glycogen metabolism were more active in the high-CRRGPI group (Fig. 6D).

### The relationship between the CRRGPI and the seven steps of antitumour immunity

**Figure 7:**
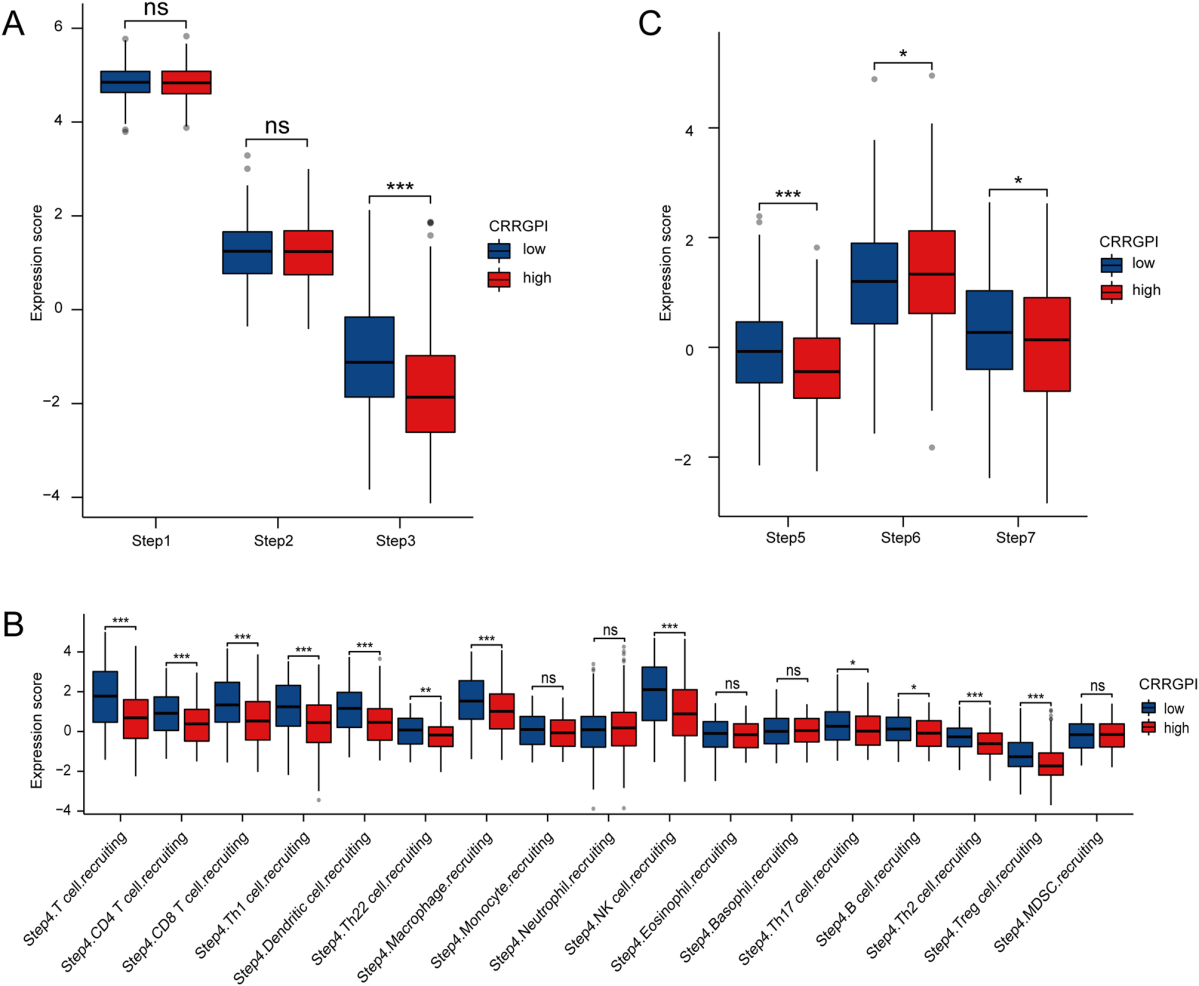
Correlation between scores of the seven-step cancer-immunity process and different CRRGPI groups. (**A**) The quantity of release of cancer antigens (step 1 of the seven-step cancer-immunity cycle), the quantity of cancer antigen presentation (step 2), the ability of priming and activating immune cells (step 3) between the different CRRGPI groups. (**B**) The ability to traffic CD8 T cells to tumors (step 4) between the different CRRGPI groups. (**C**) The degree of infiltration of immune cells into tumors (step 5), the ability to recognize cancer cells by T cells (step 6), the ability to kill cancer cells (step 7) between the different CRRGPI groups.

The anticancer immune response can be conceptualized as a series of events, including the release of cancer antigens (step 1), cancer antigen presentation (step 2), initiation and activation (step 3), transportation of immune cells to the tumour (step 4), immune cell infiltration of the tumour (step 5), T-cell recognition of cancer cells (step 6) and killing of cancer cells (step 7)^20^. These processes not only reflect the process of tumour recognition and killing but also affect the results of cancer immunotherapy. To explore the impact of the CRRGPI score on 28 immune cell types in the seven steps of the antitumour response, we downloaded the characteristic genes of the seven steps from the TIP database (supplementary Table 2) and analysed the characteristics of each process. Subsequently, we compared the seven antitumour steps of patients in different CRRGPI groups using a nonparametric test. As shown in Fig. 7, there was no significant difference in the ability to release and present tumour antibodies (steps 1 and 2) (Fig. 7A). However, compared to the high-CRRGPI group, the initiating and activating abilities of immune cells in the low-CRRGPI group were significantly higher (step 3) (*P* < 0.001) (Fig. 7A), and the total chemotaxis of the low-CRRGPI group was significantly better than that of the high-CRRGPI group (step 4) (Fig. 7B). Specifically, the chemotactic ability of CD4 T cells, CD8 T cells, Th1 cells, dendritic cells, Th22 cells, macrophage cells, NK cells, Th17 cells, B cells, Th2 cells, and Treg cells in the low-CRRGPI group was stronger than that in the high-CRRGPI group. Next, in terms of the ability of immune cells to infiltrate tumours (step 5) (Fig. 7C), patients in the low-CRRGPI group were significantly stronger (*P* < 0.001). However, in the process by which T cells recognize cancer cells, the ability of low-CRRGPI patients was significantly lower than that of high-CRRGPI patients (step 6) (*P* < 0.05) (Fig. 7C), and low-CRRGPI patients were superior to high-CRRGPI patients in terms of the ability to kill tumour cells (step 7) (*P* < 0.05) (Fig. 7C).

### Characteristics of the tumour immune microenvironment in different CRRGPI groups

**Figure 8:**
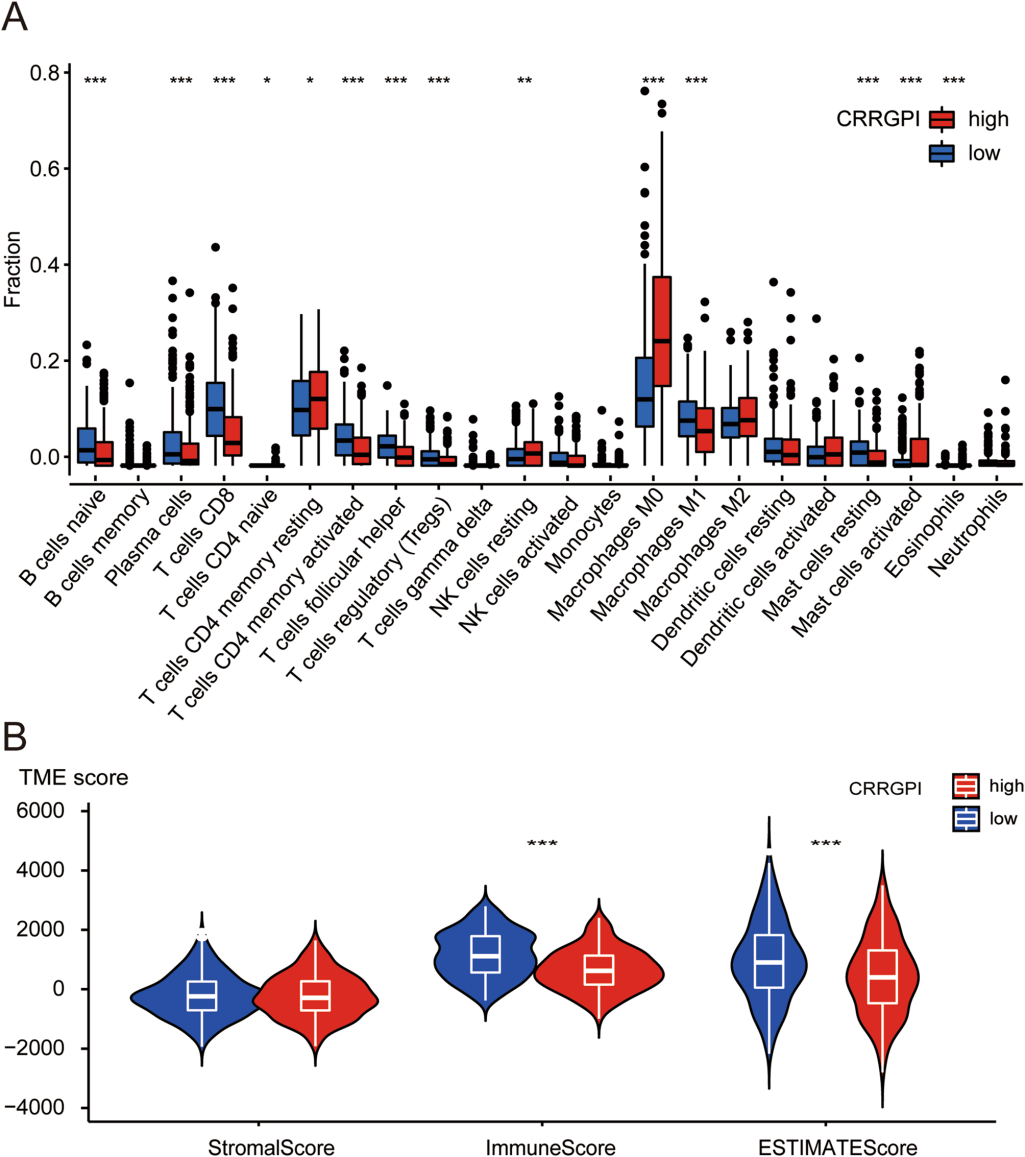
Characteristics of the tumour immune microenvironment in different CRRGPI groups. (**A**) The CIBERSORT algorithm was used to compute the score levels of infiltration of 22 immune cells. (**B**) The Stromal Score, Immune Score, and ESTIMATE Score (tumor purity) of the two CRRGPI groups. (*P < 0.05; **P < 0.01; ***P < 0.001).

To compare the immune characteristics of patients with different CRRGPI scores, we used the CIBERSORT algorithm to comprehensively compare the enrichment of 22 immune-infiltrating cells in the two CRRGPI groups. As shown in the figure, we found higher enrichment levels of naive B cells, plasma cells, CD8 T cells, memory-activated CD4 T cells, follicular helper T cells, Treg cells, activated NK cells, M1 macrophages, resting dendritic cells, and resting mast cells in the low-CRRGPI group, but the enrichment levels of memory resting CD4 T cells, resting NK cells, M0 macrophages, M2 macrophages, and activated dendritic cells were higher in the high-CRRGPI group (Fig. 8A) (*P* < 0.05). Moreover, we also evaluated the tumour microenvironment components of different groups by using the ESTIMATE algorithm. Finally, we calculated the ESTIMATE score, immune score, and stromal score in each patient sample to compare the composition characteristics of the tumour microenvironment. The ESTIMATE score is the sum of the immune score and stromal score, which can reflect the ratio of immune cells to stromal cells in the patient's microenvironment and predict tumour purity. The higher the ESTIMATE score was, the higher the ratio of the immune score to the stromal score, which suggested that the proportion of tumour cells was low. Our results showed that both the ESTIMATE score and immune score of HNSCC patients with high CRRGPI were lower than those of HNSCC patients with low CRRGPI (*P* < 0.001), while there was no significant difference in stromal score between the two groups (Fig. 8B), which was similar to the results from the CIBERSORT algorithm.

### Benefits of immune checkpoint inhibitors in different groups

Since the CRRGPI can distinguish the OS of HNSCC patients and is related to multiple antitumour immune response processes, we speculated that the CRRGPI may be used to predict which patients could benefit from ICIs.

First, we used correlation analysis to evaluate the correlation between the CRRGPI and some immune checkpoint-related genes, including *PDCD1* (PD1), *CD274* (PD-L1), and *CTLA4*. The results showed that the CRRGPI was negatively correlated with commonly used immune checkpoint-related genes (Supplementary Fig. 4), and the expression levels of PD1, PD-L1, and CTLA4 in patients in the low-CRRGPI group were higher than those in the high-CRRGPI group. Many studies have shown that the higher the expression of immune checkpoint proteins, such as PD1, PD-L1, and CTLA4, the greater the clinical benefit for patients with carcinoma from ICIs. In summary, we predicted that patients in the low-CRRGPI group were more likely to benefit from ICIs.

**Figure 9:**
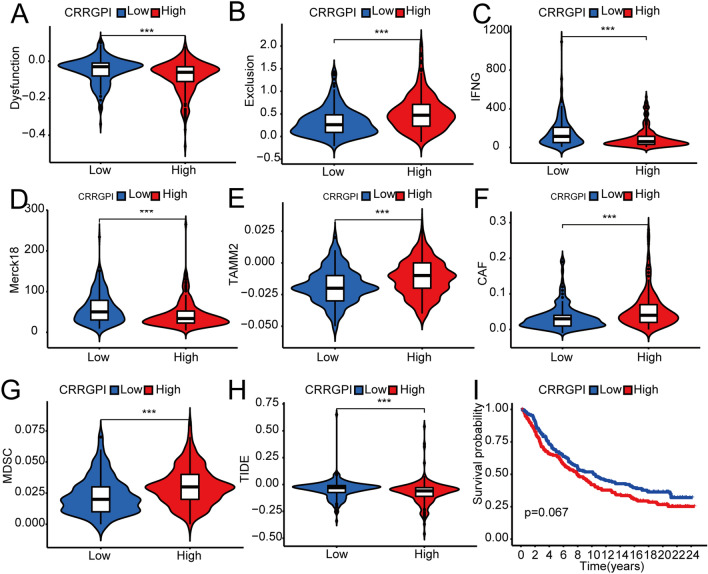
Predicting the response to immunotherapy in patients with different CRRGPI scores. (**A**–**H**) T-cell dysfunction score, T-cell exclusion score, IFNG, Merck18, M2-TAMs, CAFs, MDSCs, and TIDE score in the different CRRGPI groups. (**I**) Kaplan–Meier analysis of different CRRGPI groups in the IMvigor210 cohort. (*P < 0.05; **P < 0.01; ***P < 0.001). CRRGPI, Circadian Rhythm-Related Gene Prognostic Index.

We then used the TIDE algorithm to further evaluate the clinical benefits of ICIs in different subgroups. We found that patients with a low CRRGPI score had higher T-cell dysfunction scores, while patients with a high CRRGPI score had higher T-cell exclusion scores (Fig. 9A,B) (*P* < 0.001). Moreover, IFNG and Merck18 (Fig. 9C,D), which can reflect the ability to release IFN-γ, showed that patients with low CRRGPI scores could release more IFN-γ, which indicated a stronger ability to kill the tumour (*P* < 0.001). In addition, the levels of three types of suppressor cells that facilitate tumour immune escape, M2-TAMs, CAFs, and MDSCs, suggested that immune escape is more likely to occur in patients with high CRRGPI scores (Fig. 9E–G). Finally, the TIDE score was obtained by comprehensive evaluation of the above results. The higher the TIDE score was, the stronger the ability to escape from immune surveillance, which means that these patients may not benefit from immunotherapy. In our results, the TIDE score of patients with low CRRGPI scores was significantly higher than that of the high-CRRGPI group (*P* < 0.001), meaning that patients with low CRRGPI scores were less likely to benefit from ICIs (Fig. 9H). Because the immunotherapy efficacy predicted by the TIDE score is not consistent with that predicted by the antitumour steps, we further used the clinical cohort to evaluate the ability of the CRRGPI to predict the effect of immunotherapy in different CRRGPI groups. In the IMvigor210 cohort of bladder cancer, patients with low CRRGPI scores demonstrated a better overall survival trend (Fig. 9I) (*P* = 0.067). According to the previous results, we predict that patients in the low-CRRGPI group may experience clinical benefits when treated with ICIs.

### Correlation between the CRRGPI and drug sensitivity

**Figure 10:**
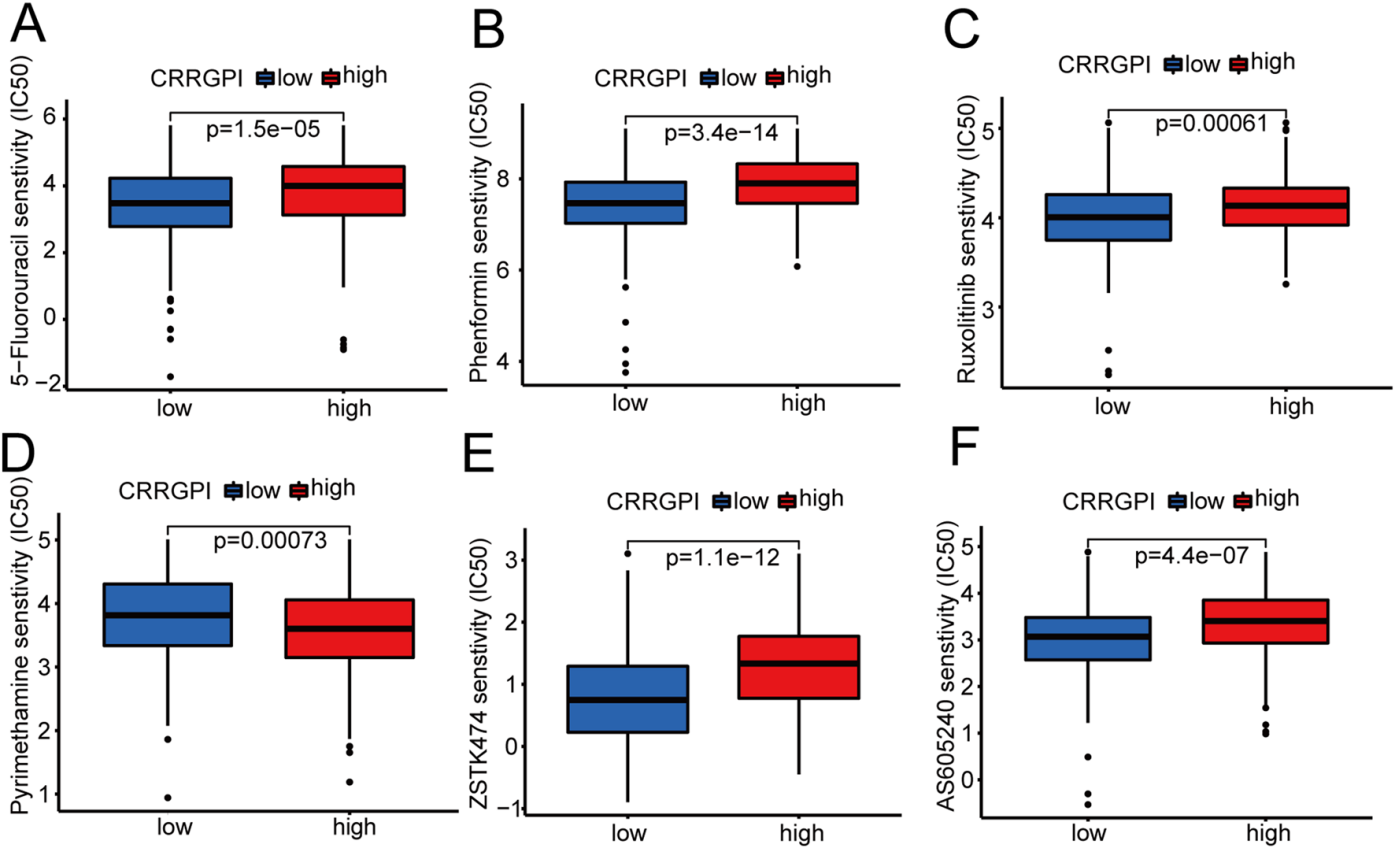
Correlation between the CRRGPI and drug sensitivity. The IC50 values for (**A**) 5-Fluorouracil (**B**) Phenformin (**C**) Ruxolitinib (**D**) Pyrimethamin (**E**) ZSTK474 (**F**) AS-605240 in the high-CRRGPI and low-CRRGPI groups.

IC50, the 50% inhibitory concentration, represents the concentration of a drug that is required for 50% inhibition of tumour cells and is commonly used as a measure of drug sensitivity. That is, the lower the IC50 value of a drug in tumour cells, the more sensitive the tumour cells are to this drug. To explore the potential chemotherapeutic drugs that might be used to treat HNSCC based on the IC50 data, we used the pRRophetic package to analyse the correlation between each patient's CRRGPI and multiple chemotherapeutic drugs’ IC50 values and explored the drug sensitivity of patients in the high- and low-CRRGPI groups. We observed that 28 drugs had lower IC50 values in the low-CRRGPI group than in the high-CRRGPI group, meaning that patients with low CRRGPI scores are more sensitive to these drugs, which included 5-fluorouracil, ZSTK474, pyrimethamine, ruxolitinib, phenformin, and AS-605240 (Fig. 10A–F). 5-Fluorouracil is a chemotherapeutic drug that can be used to treat HNSCC. ZSTK474 and AS-605240 are both PI3K inhibitors. Sho et al.^21^ reported that a PI3K inhibitor in combination with immune checkpoint blockade can inhibit Tregs and generate memory CD8 + T cells, resulting in durable antitumour immunity. Ruxolitinib, a selective inhibitor of Janus kinase (JAK) 1 and 2, can provide significant clinical benefits in patients with myelofibrosis and improve OS^22^. Phenformin, a derivative of metformin with higher anticancer potency at lower doses, can effectively inhibit bladder cancer growth by activating AMPK signalling and inhibiting EGFR signalling^23^. Concurrently, we found that the high-CRRGPI group was more sensitive to pyrimethamine. Pyrimethamine, a subclass of antifolate drugs, can block dihydrofolate reductase. Several studies have reported that pyrimethamine can induce antitumour immune effects by various mechanisms, such as the induction of cathepsin B-dependent and caspase-dependent apoptotic pathways and the inhibition of STAT3^24^.

In addition, we used the CellMiner database to analyse the correlation between the expression of the 9 circadian rhythm genes and drug sensitivity. First, the gene expression level of 60 patients with carcinoma and the sensitivity data of 216 clinical drugs approved by the Food and Drug Administration (FDA) were downloaded from the CellMiner database. Then, Pearson correlation analysis was used to obtain the correlation between the 9 circadian rhythm genes and drug sensitivity. The results are shown in Supplementary Fig. 5 (Cor > 0.5, *P* < 0.05).

## Discussion

HNSCC has a high incidence and poor prognosis. At present, there are few prognostic markers used to guide the individualized treatment regimens of patients with HNSCC. Moreover, rates of local recurrence after standard chemoradiotherapy remain high, and the efficacy of immune checkpoint therapy has been limited. Therefore, there is an urgent need for an indicator to predict prognosis and guide the personalized treatment of patients with HNSCC. An increasing number of studies have shown that circadian rhythm plays an important role in tumour development and progression and affects the prognosis of patients. A clinical study showed that the prognosis and treatment-related toxicity of patients with HNSCC were correlated with the time to receive radiotherapy^7,25^. The study’s results further showed that the prognosis of patients may be affected by circadian rhythm and that the characteristics of circadian rhythm may be used to guide the delivery time of antineoplastic therapy. However, there are few studies on the impact of circadian rhythm-related genes on HNSCC.

In this study, we constructed a prognostic signature consisting of 9 circadian rhythm-related genes (*ADA*, *ICOS*, *ALG3*, *STC1*, *CYP4X1*, *EZH2*, *OLR1*, *PLEKHA6*, and *STARD4*) to predict the prognosis of HNSCC patients. Internal verification with TCGA data and external verification with GEO data showed that the prognostic signature had high reliability.

Altered ADA activity is thought to be associated with various kinds of tumours. Elevated ADA activity promotes the progression of breast cancer, colorectal cancer, gastric cancer, renal cell carcinoma, and other tumours^26^. However, some studies have reported that low ADA activity can promote tumour progression in head and neck tumours. Additionally, the ADA/CD26 levels of exosomes and T cells are relatively normal in the early stage of the disease, but ADA and CD26 are significantly higher in the CD3 fraction of exosomes in patients with low-stage HNSCC^27^. Currently, given the multiple biological functions of ADA, some ADA inhibitors have been used to treat the rare haematologic malignancy of hair-cell leukaemia^28^. ICOS, a T-cell immune-costimulatory receptor, regulates tumour immunity via two different mechanisms. On the one hand, ICOS can promote the activation of cytotoxic T-cell-mediated antitumour immunity. On the other hand, ICOS promotes the survival of Treg cells, thereby weakening antitumour immunity^29^. Treg cells are highly expressed in the tumour microenvironment of most cancers and lead to poor patient prognosis. For example, compared with early-stage gastric cancer, a higher proportion of Tregs express ICOS in advanced gastric cancer^30^. Since the ICOS expression level and ICOS/ICOS-L pathway play an important role in antitumour immunity, they are hypothesized to be biomarkers for immunotherapy. ALG3, a-1,3-mannosyl transferase, is involved in protein glycosylation during endoplasmic reprogramming. A high ALG3 expression level may promote tumour proliferation, metastasis, and metabolic reprogramming. It is associated with poor prognosis in patients with HNSCC^31,32^. Sun et al. found that ALG3 can regulate the radiosensitivity of breast cancer by regulating the glycosylation of TGFBR2^33^. In addition, ALG3 modulates tumour immunogenicity through various pathways and is believed to be a therapeutic target to enhance the efficacy of ICIs^34^. STC1 expression in a tumour can promote tumour metastasis and invasion through different mechanisms. For example, STC1 promotes ovarian cancer metastasis, lipid metabolism, and DDP chemotherapy resistance through the FOXC2/ITGB6 signalling pathway^35^. Yan et al. showed that the malignant biological behaviour of glioma is related to the TGF-β/SMAD4 signalling pathway mediated by STC1^36^. Moreover, STC1 expressed in tumour cells promotes immune escape and resistance to immunotherapy by inhibiting APC phagocytosis^37^. Therefore, patients may obtain greater clinical benefits from immunotherapy by targeting STC1. There are few studies on the role of CYP4X1 in tumour occurrence and development. Chen long Wang et al. found that inhibition of CYP4X1 in TAMs can prolong the survival time of glioma patients and normalize tumour blood vessels through the CB2 and EGFR-STAT3 signalling pathways^38^. Therefore, targeting CYP4X1 may be a novel treatment strategy for carcinoma. In addition, CYP4X1 affects the efficacy of chemotherapy. Gastric cancer patients with low expression of CYP4X1 receive less clinical benefit from capecitabine and cisplatin and have a worse survival prognosis^39^. At present, the role of CYP4X1 in HNSCC has not been reported. EZH2 is a tumour suppressor in OSCC. Studies have reported that EZH2 knockout in the oral basal epithelium can lead to the development of OSCC, so EZH2 may be a strong candidate for designing therapeutic strategies for OSCC patients^40^. OLR1 is a human gene encoding oxidized low-density lipoprotein receptor 1 (OLR1). Macrophage-associated OLR1 is significantly expressed in HNSCC and may be involved in the formation of an immunosuppressive tumour microenvironment. In addition, OLR1 may be involved in reprogramming the lipid metabolism of tumour cells in HNSCC, thereby altering the tumour immune microenvironment and affecting the prognosis of patients^41,42^. PLEKHA6 is a member of the PLEKHA protein family, and few studies on PLEKHA6 have been reported. STARD4, a cholesterol transporter, is involved in the regulation of intracellular cholesterol homeostasis^43^. Some researchers have reported that STARD4 is highly expressed in breast cancer tissues and promotes tumour progression by regulating cholesterol metabolism. It has also been clinically observed that patients with high expression of STARD4 have shorter metastasis-free survival^44^. In conclusion, all these genes participate in the occurrence and development of tumours via various mechanisms, which further explains why the CRRGPI can be an independent prognostic factor for HNSCC. Interestingly, most of the circadian rhythm genes in the prognostic signature are involved in regulating immune cell activity and metabolic reprogramming, thus forming a microenvironment promoting tumour growth. Therefore, it is necessary to analyse the immune microenvironment and metabolic characteristics of patients with different CRRGPI scores, which will help us further understand how circadian rhythm affects tumorigenesis and tumour progression.

To further understand the characteristics of patients with different CRRGPI scores in terms of biological function, the KEGG and GO databases were used to analyse the DEGs between different CRRGPI groups. First, according to the KEGG database, we found that most of the DEGs are involved in metabolic pathways, such as purine metabolism; fatty acid metabolism; glycolysis/gluconeogenesis; bile secretion; and valine, leucine, and isoleucine degradation. In addition, cellular components (CCs), molecular functions (MFs), and biological processes (BPs) were further analysed based on the GO database. Most of the DEGs were concentrated in immunity, such as the B-cell receptor signalling pathway, antigen binding, immunoglobulin receptor binding, and immunoglobulin complex. Thus, we speculate that there are differences in metabolism and immune function between the two CRRGPI groups. Next, to further investigate the metabolic characteristics of the different CRRGPI groups, we evaluated their scores on some metabolic pathways. The metabolic scores showed that compared to the low-CRRGPI group, the purine metabolism, glycerolipid metabolism, and glycogen metabolism scores were higher in the high-CRRGPI group. Some studies have shown that purines are the most elementary metabolic substrates of all organisms, providing essential components for DNA and RNA synthesis, including in tumour cells. Purine nucleotide metabolism is an important component in the DNA synthesis process and high proliferative activity of tumour cells, so purine and purine biosynthesis pathways are essential for tumour cell proliferation. In addition, purine can regulate the immune cell response and cytokine release through various receptor subtypes, which play an important role in tumorigenesis^45^. Glycogen is the primary source of carbohydrate storage in mammals. However, an abnormal increase in the amount of glycogen has been observed in many cancers, such as lung cancer, breast cancer, and kidney cancer^46^. Recent studies have shown that glycogen metabolism affects immune functions and that inhibition of glycogenolysis significantly attenuates TLR-mediated DC maturation and impairs their ability to initiate lymphocyte activation, thereby regulating their optimal immune function^47^. These results suggest that hyperactive cell metabolism may be associated with tumours.

To further understand the mutation signature of different CRRGPI groups, we explored the TMB. Although the results showed no significant difference in TMB between the high-CRRGPI and low-CRRGPI groups, we observed that the mutation frequency of TP53 was higher in high-CRRGPI patients (80%) than in low-CRRGPI patients (59%). Previous studies have shown that TP53, a tumour suppressor gene, can inhibit cell proliferation and promote cell apoptosis, thus exerting an antitumour effect. HNSCC patients with TP53 mutations have worse OS^48–50^. In our study, patients with high CRRGPI had worse OS, which may be related to the high frequency of TP53 mutations. Since most of the genes in the CRRGPI are involved in immunoregulation, we further explored the relationship between the CRRGPI and antitumour immune processes, the immune tumour microenvironment, and infiltrating immune cells. In the processes of antitumour immunity, our results showed that the activities of immune cells, chemotaxis of immune cells to tumour tissues, and infiltration degree of immune cells in the low-CRRGPI group were significantly higher than those in the high-CRRGPI group, including CD4 + T cells, CD8 + T cells and NK cells, which play a major role in killing tumour cells. In conclusion, patients with low CRRGPI scores have stronger activation and transport capacity of immune cells and greater immune cell infiltration, thus generating stronger antitumour immune responses and improving the outcomes of patients with HNSCC. This explains the correlation between the CRRGPI and the outcome of HNSCC patients.

Given the good predictive effect of the CRRGPI in the traditional treatment cohorts, such as the radiotherapy cohort and targeted therapy cohort, as well as the relationship between the CRRGPI and the antitumour immune response, we used the TIDE algorithm to evaluate the predictive ability of the CRRGPI in the therapeutic efficacy of ICIs. Using the T-cell dysfunction score and exclusion score, the TIDE algorithm can be used to evaluate immune evasion capacity and predict the benefits of ICIs. TIDE algorithm assessment showed that T-cell exclusion scores, CAFs, MDSCs, and M2-TAMs in the low-CRRGPI group were lower than those in the high-CRRGPI group. Conversely, patients with a low CRRGPI had a higher T-cell dysfunction score. Next, the T-cell dysfunction score and T-cell exclusion score were comprehensively evaluated to obtain the TIDE score. The higher the TIDE score was, the lower the clinical benefit from ICIs. However, to our surprise, the TIDE score was higher in the low-CRRGPI group than in the high-CRRGPI group, indicating that patients with low-CRRGPI had a stronger ability of tumour immune escape, which implies a worse outcome in the low-CRRGPI group when treated with ICIs. This conclusion is not consistent with the previous results.

Therefore, to further confirm the prognostic value of the CRRGPI, survival analysis was performed on the clinical cohort treated by ICIs according to the characteristics of the CRRGPI. The results showed that patients with low CRRGPI scores were more likely to benefit from immunotherapy and had a better OS than those with high CRRGPI scores.

In conclusion, the CRRGPI can be used to comprehensively evaluate the prognosis and biological characteristics of HNSCC patients, including metabolism, tumour stemness, and the immune microenvironment, and predict the potential immunotherapy response. Moreover, the CRRGPI may be a prognostic marker to guide individualized treatment regimens for HNSCC.

### Supplementary Information

Supplementary Legends.

Supplementary Figure 1.

Supplementary Figure 2.

Supplementary Figure 3.

Supplementary Figure 4.

Supplementary Figure 5.

Supplementary Table 1.

Supplementary Table 2.

## Data Availability

The TCGA (The Cancer Genome Atlas) dataset is available using the following link: https://portal.gdc.cancer.gov/. The GEO (Gene Expression Omnibus) dataset is available using the following link: https://www.ncbi.nlm.nih.gov/geo/.
