# Supplementary material for: A signature based on circadian rhythm-associated genes for the evaluation of prognosis and the tumour microenvironment in HNSCC
Source: Sci Rep. 2024 Mar 31;14:7594. doi: 10.1038/s41598-024-57160-5 (PMC10982303; doi:10.1038/s41598-024-57160-5)
Supplement: Supplementary file 1 — Supplementary Legends. [file 41598_2024_57160_MOESM1_ESM.docx]

**Supplementary figure legends**

**Supplementary Fig1. Assessment of CRRGPI as Independent HNSCC prognostic factors for PFS.** The forest map shows the CRRGPI and the clinical variables by univariate Cox regression analysis (A) and multivariate Cox regression analysis (B).

**Supplementary Fig2. The predictive performance of CRRGPI for overall survival in HNSCC.** (A) The receiver operating characteristic (ROC) curves of the CRRGPI of patients in the GEO cohort; (B) The receiver operating characteristic (ROC) curves of CRRGPI, TIDE, and TIS of patients in the TCGA cohort.

**Supplementary Fig 3. Subgroup survival analysis.** Kaplan‒Meier plots of high-CRRGPI and low-CRRGPI subtypes of HNSCC in 6 subgroups of (A) patients aged <65; (B) patients aged ≥65; (C) female patients; and (D) male patients.

**Supplementary Fig 4.** (A) Correlation between the CRRGPI and immune checkpoint genes. Red represents a positive correlation, and blue represents a negative correlation. (B-D) Comparisons of CD274, PDCD1, and CTLA-4 between the high-CRRGPI group and the low-CRRGPI group. (* p<0.05)

**Supplementary Fig 5.** The correlation between the 9 circadian rhythm genes and drug sensitivity. (Cor>0.5, P<0.05)
