# Supplementary figures and images for: A signature based on circadian rhythm-associated genes for the evaluation of prognosis and the tumour microenvironment in HNSCC
Source: Sci Rep. 2024 Mar 31;14:7594. doi: 10.1038/s41598-024-57160-5 (PMC10982303; doi:10.1038/s41598-024-57160-5)

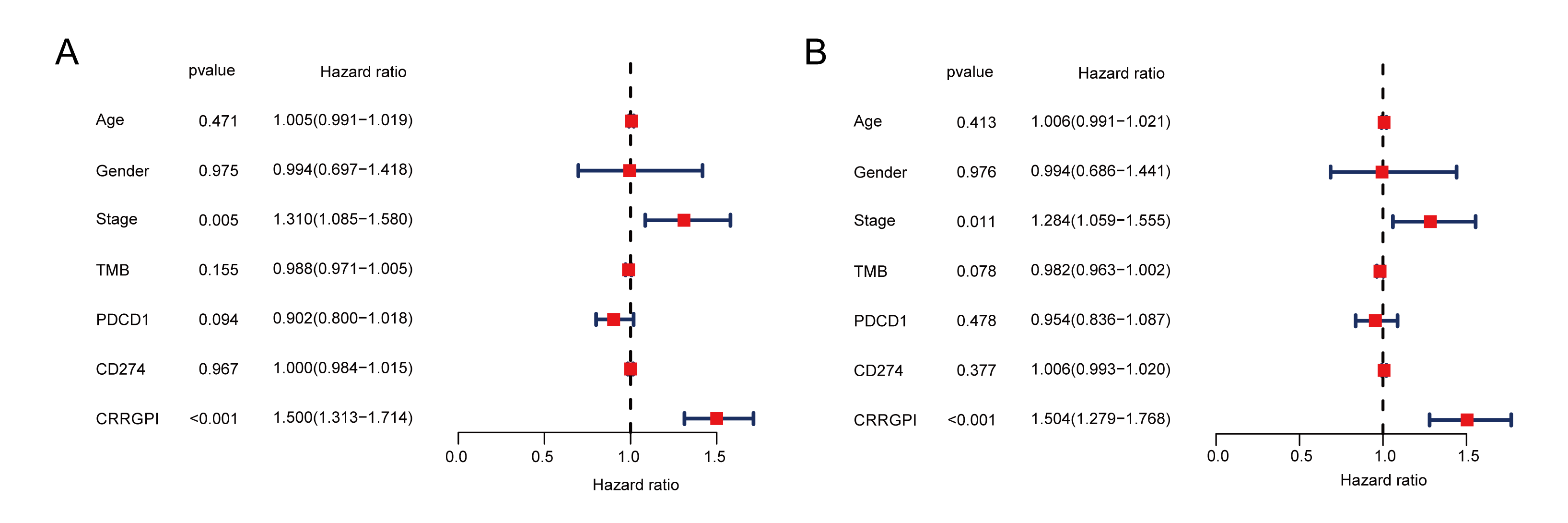

Supplement: Supplementary file 2 — Supplementary Figure 1. [file 41598_2024_57160_MOESM2_ESM.tif]

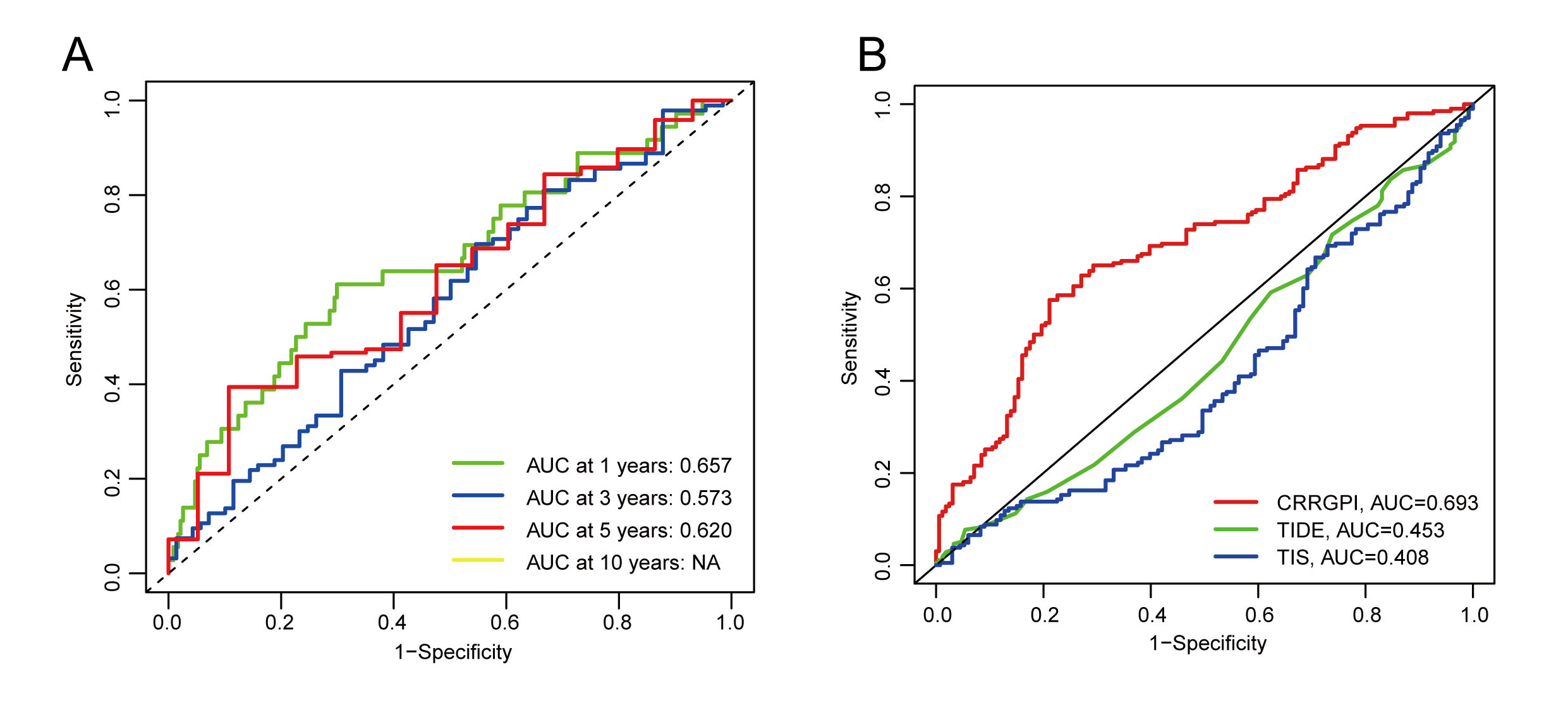

Supplement: Supplementary file 3 — Supplementary Figure 2. [file 41598_2024_57160_MOESM3_ESM.tif]

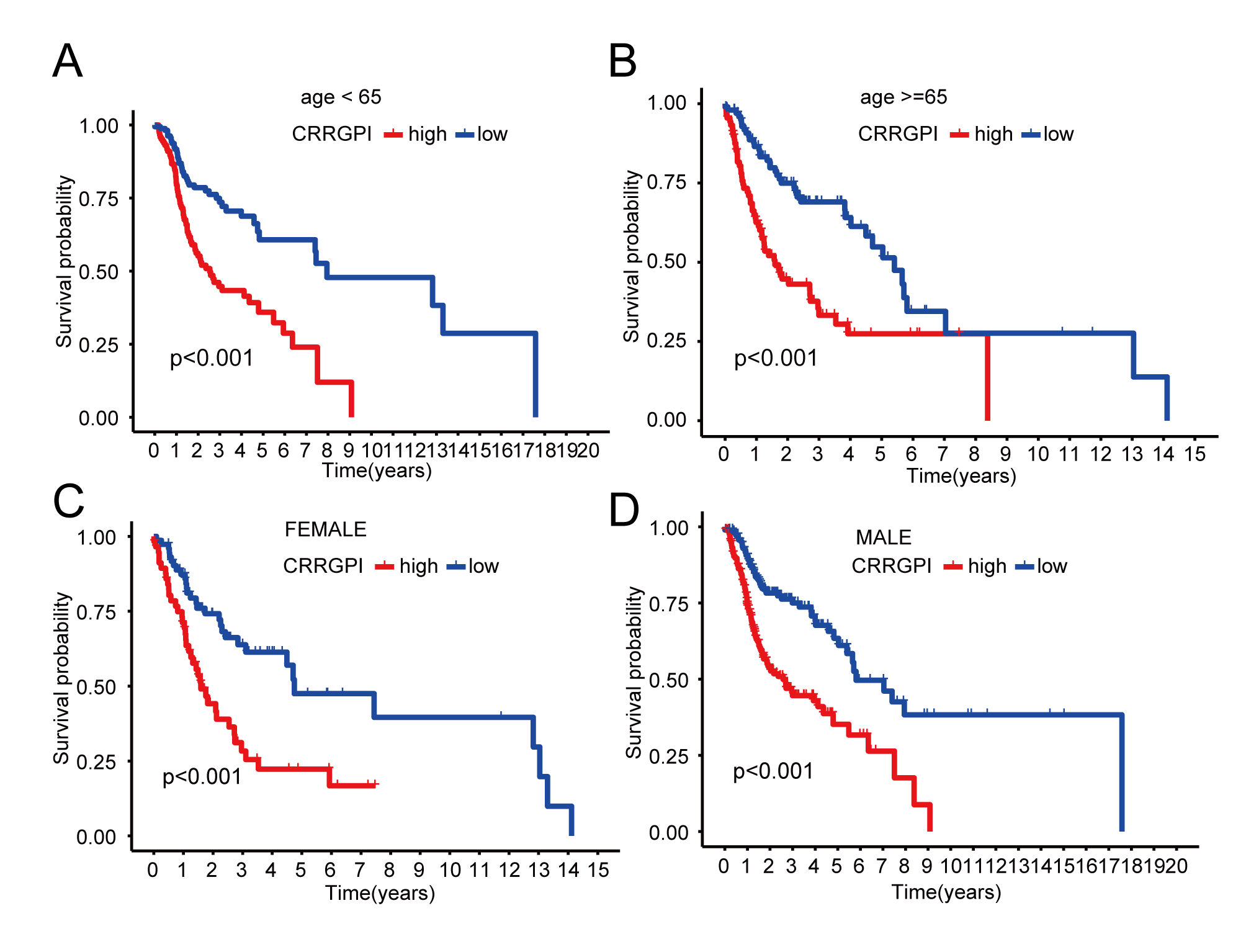

Supplement: Supplementary file 4 — Supplementary Figure 3. [file 41598_2024_57160_MOESM4_ESM.tif]

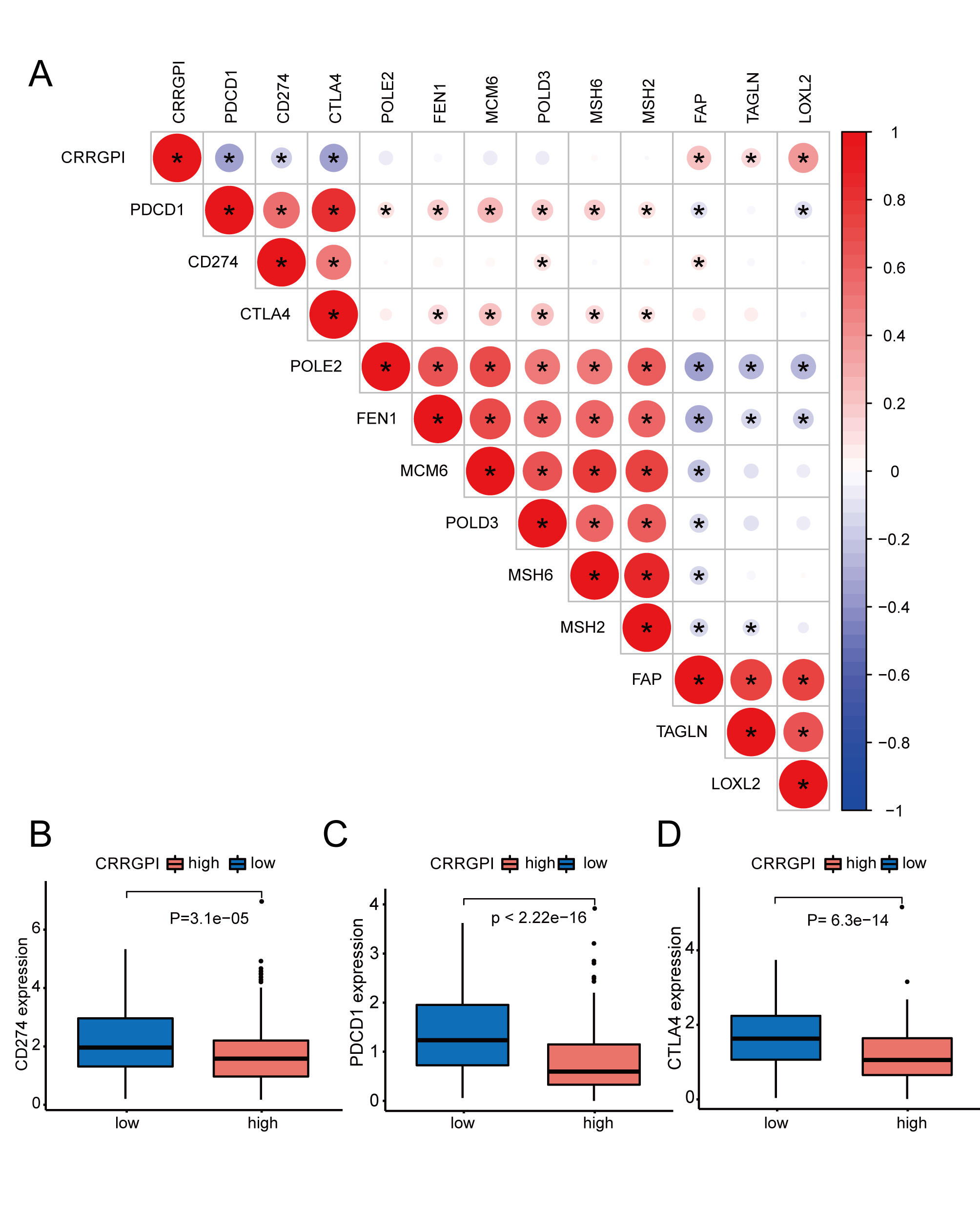

Supplement: Supplementary file 5 — Supplementary Figure 4. [file 41598_2024_57160_MOESM5_ESM.tif]

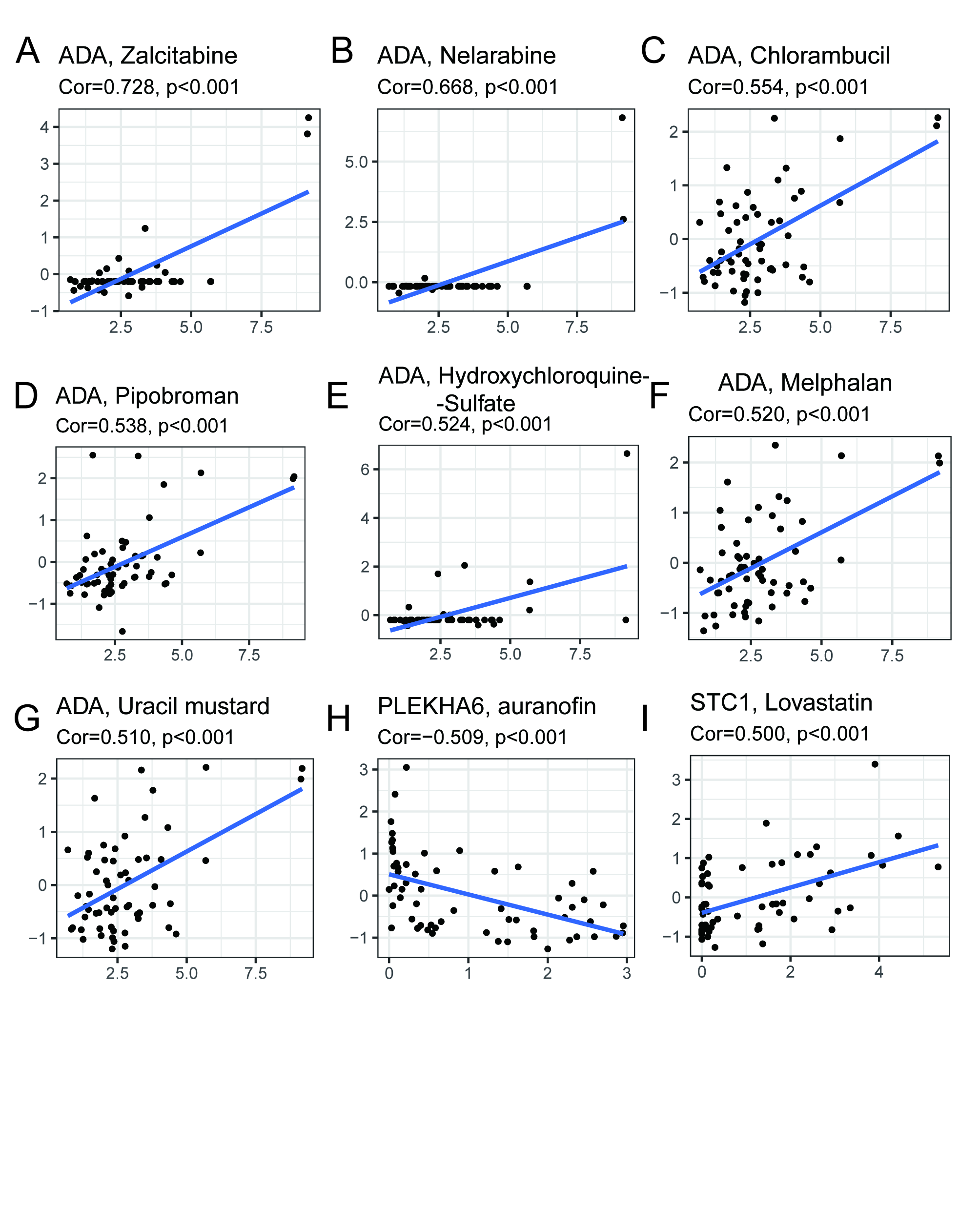

Supplement: Supplementary file 6 — Supplementary Figure 5. [file 41598_2024_57160_MOESM6_ESM.tif]
